# Daytime Mouthpiece Ventilation Combined with Nocturnal Non-Invasive Ventilation in Adults with Duchenne Muscular Dystrophy: A 30-Day Pilot Feasibility Study

**DOI:** 10.3390/jcm15218636

**Published:** 2026-11-05

**Authors:** Anna Annunziata, Angela Irene Mirizzi, Antonietta Coppola, Maria Rosaria Valentino, Rosa Saggio, Pasqualina Gaglione, Gianfranco Gaglione, Gennaro Calafiore, Cristina Liguori, Giuseppe Fiorentino

**Affiliations:** 1 Department of Respiratory Physiopathology, AORN Ospedali dei Colli-Monaldi, 80131 Naples, Italy; angela.mirizzi1989@gmail.com (A.I.M.); antonietta.coppola84@gmail.com (A.C.); rosyvalentinodoc@gmail.com (M.R.V.); dottssa.pasqualinagaglione@gmail.com (P.G.); gianfrancogaglione@libero.it (G.G.); 2 NEMO Clinical Center, AORN Ospedali dei Colli-Monaldi, 80131 Naples, Italy; rosa.saggio94@gmail.com (R.S.); ge.calafiore@gmail.com (G.C.); cristina.liguori@centrocliniconemo.it (C.L.); 3 U.O.C. of Pneumology and Respiratory Semi-Intensive Care, Federico II University Hospital, 80131 Naples, Italy; giuseppe.fiorentino3@unina.it

**Keywords:** Duchenne muscular dystrophy, mouthpiece ventilation, non-invasive ventilation, respiratory failure, home mechanical ventilation, neuromuscular disease, S3-NIV

## Abstract

**Background:** DMD causes progressive respiratory muscle weakness and chronic alveolar hypoventilation requiring ventilatory support. While nocturnal non-invasive ventilation (NIV) is the standard care, daytime ventilation is often provided with prolonged use of nasal–oronasal masks. These interfaces can cause skin injury, air leaks, and reduced adherence. Mouthpiece ventilation (MPV) provides on-demand pressure support without an interface and may be an alternative for intermittent daytime ventilation. **Objective**: To assess the feasibility, safety, and clinical impact of MPV as an adjunct to nocturnal NIV in patients with DMD. **Methods**: Six patients with DMD and chronic respiratory failure on nocturnal NIV were enrolled in a 30-day observational pilot study. After training, participants used MPV during daytime. Clinical assessment, arterial blood gases, and quality of life evaluated with the S3-NIV questionnaire were performed at baseline (T0) and after 30 days (T1). **Results**: All patients tolerated MPV well, with mean daily use of >16 h. PaCO_2_ decreased in all patients (from 49.7 ± 7.1 to 43.5 ± 5.7 mmHg; *p* = 0.031); 4/6 were normocapnic at T1, whereas hypercapnia persisted in two. S3-NIV scores improved from 2.53 ± 0.69 at T0 to 4.87 ± 0.91 at T1 (*p* = 0.031). Patients reported improved performance of daily activities and better psychological well-being. No MPV-related adverse events occurred. **Conclusions**: MPV was feasible, safe, and well tolerated as an adjunct to nocturnal NIV in this small series of patients with DMD over 30 days. Given the small sample and short follow-up, no firm conclusions can be drawn; larger studies with longer follow-up are needed to confirm these findings and define the optimal role of MPV in DMD respiratory management.

## 1. Introduction

Respiratory failure and nocturnal hypoventilation are major complications in patients with Duchenne muscular dystrophy (DMD). DMD has a worldwide prevalence of approximately 7.1 per 100,000 males [1]. In a recent Italian nationwide survey of all 31 reference centres, 972 patients with DMD were identified, 41% of whom were adults; approximately 10% used ventilatory support for more than 12 h/day and 1% had a tracheostomy [2]. As survival improves, the number of young adults requiring prolonged daytime ventilatory support is expected to increase. Although non-invasive ventilation (NIV) significantly improves survival rates [3,4], conventional interfaces—such as nasal or oronasal masks—are often associated with poor patient compliance because of claustrophobia, skin breakdown, and air leaks, especially in patients who require frequent daytime NIV sessions [5,6]. Other daytime NIV procedures should be considered in highly ventilator-dependent patients in addition to mask ventilation [7,8,9]. Mouthpiece ventilation (MPV) is non-invasive ventilation delivered via a mouthpiece. MPV was first used in ventilator-dependent polio patients [10,11].

In recent years, mouthpiece ventilation modes have been introduced into commercially available portable ventilators, increasing interest in this ancient–modern interface [5,6]. Mouthpieces are useful as an adjunct for daytime ventilation for patients with neuromuscular diseases who cannot maintain acceptable diurnal arterial blood gases without frequent, intermittent assistance [12]. Khirani et al. evaluated ventilators for mouthpiece ventilation in patients with neuromuscular disease and found that subjects were satisfied with this interface [13].

The use of mouthpiece ventilation in patients with Duchenne muscular dystrophy is documented in a few studies [3,14]. McKim et al. suggested that twenty-four-hour NIV should be considered a safe alternative for patients with DMD, as it may obviate the need for tracheostomy in patients with chronic respiratory failure requiring more than nocturnal ventilation alone [15]. As the disease progresses, patients with DMD develop chronic hypoventilation and may require respiratory support 24 h a day. In this context, mouthpiece ventilation can be particularly valuable for patients who use NIV for many hours a day and develop skin lesions, gastric distension, or eye irritation from alternating nasal and full-face masks. By preserving speech and swallowing, MPV is particularly suitable for young adults with DMD who require prolonged ventilatory support but wish to maintain communication and social interaction [5,16].

Toussaint et al. evaluated the addition of MPV as an alternative to 24 h NIV in 42 consecutive end-stage DMD patients who were normocapnic at night under NIV but showed daytime hypercapnia. Diurnal MPV was delivered via an angled mouthpiece held by a specialized self-supporting shoulder harness [17]. They concluded that daytime mouthpiece ventilation is a safe, highly effective alternative to invasive tracheostomy for end-stage DMD patients.

In our centre, daytime MPV was well tolerated by four young male patients with severe restrictive respiratory patterns (FVC ≤ 1 L) and a history of NIV non-compliance, and enabled two of them to accept nocturnal nasal-mask NIV [18]; similarly, in a patient with DMD admitted with hypercapnic respiratory failure who had previously rejected positive airway pressure therapy, MPV initiated as a rescue intervention restored blood gases and promoted adherence to nocturnal NIV [19].

Despite this evidence, knowledge and practical use of MPV remain limited in many centres, even though an international expert workshop has issued best-practice recommendations [6].

The present study builds directly on this published experience and on our centre’s previous experience with MPV in DMD [18,19]. Unlike the larger series by McKim et al., Toussaint et al. and Bach and Martinez, in which MPV was introduced in end-stage patients to extend ventilation into the daytime once diurnal hypercapnia had developed [3,15,17], the present study addresses patients who were already ventilated for more than 16 h/day and well adapted to nocturnal NIV, but who had become intolerant of daytime mask ventilation because of facial pressure ulcers or interference with social life; in these patients, MPV was used to replace daytime mask ventilation rather than to extend ventilatory support, and its short-term effects were assessed with arterial blood gases and a patient-reported questionnaire.

Our study aimed to evaluate the role of MPV as an add-on to nocturnal NIV in patients with DMD. Specifically, we summarize current evidence on the safety and efficacy of MPV in DMD, describe practical implementation, and discuss its potential impact on treatment adherence and clinical outcomes.

## 2. Materials and Methods

### 2.1. Study Design and Patients

This single-centre observational pilot study was conducted from November 2024 to November 2025 at the Department of Respiratory Physiopathology and the NEMO Clinical Center, AORN Ospedale–Monaldi, Naples, Italy.

We evaluated six patients affected by Duchenne muscular dystrophy and chronic respiratory failure who complained of discomfort with NIV. They were receiving NIV via a nasal mask for more than 16 h a day, i.e., nocturnal NIV plus daytime nasal-mask ventilation (Table 1). Three patients (patients 2, 4, and 5) used an oronasal mask at night. Patients were eligible if they had DMD with chronic respiratory failure, were receiving home NIV for more than 16 h/day, and reported discomfort or complications related to daytime mask ventilation.

All the patients reported discomfort during NIV and interruptions during the day to perform activities of daily living (frequency of daytime visits to diurnal centre, meeting with friends, university, etc).

Functional parameters are listed in Table 1. Patients reported discomfort while using NIV due to nasal or facial pressure sores, a sensation of tightness during daytime use, the inability to call a family member in the next room, and the inability to interact socially (patients 3, 4, 5 and 6).

During a follow-up respiratory assessment, after evaluating the reported issues, we offered each patient a trial of MPV. All patients agreed to undergo the MPV trial.

### 2.2. MPV Set-Up and Titration

The MPV trial was conducted on an outpatient basis; settings were determined for each patient, and comfort, tolerance, and efficacy in maintaining adequate oxygen saturation targets (>94–95%) were assessed. CO_2_ levels were monitored via capnography. After the settings had been established, patients received practical training in the use of MPV during the outpatient trial.

Three patients were fitted with a circuit with an expiratory valve because they could not disconnect from the mouthpiece during exhalation. Three patients were fitted with a dedicated single-limb circuit. We mounted a dedicated rigid support arm on their wheelchairs.

Three patients were set to ST mode, starting with an IPAP of 8–10 and increasing by 2 cm H_2_O progressively until achieving optimal saturation and good chest expansion; a pressure-targeted mode was used in these patients because they were already adapted to pressure-targeted ventilation with the same ventilator during nocturnal NIV. Three patients were set to ACV mode, starting with a volume of 400–500 mL (approximately 6–10 mL/kg of ideal body weight, calculated with the Devine formula using height estimated from arm span).

### 2.3. Airway Clearance and Secretion Management

Airway secretion management, a key determinant of the success of NIV in neuromuscular patients [8,20,21], was continued unchanged throughout the study. All patients used mechanical insufflation–exsufflation at home, at least once daily and as needed during respiratory infections, combined with manually assisted cough; patients ventilated in volume mode were also trained to perform breath stacking through the mouthpiece. Patients and caregivers were instructed to intensify airway clearance in case of respiratory infection or oxygen desaturation.

### 2.4. Outcome Measures

Feasibility was assessed as completion of the 30-day period with continued daily use of MPV; safety, as the occurrence of MPV-related adverse events; and clinical impact, as the changes in arterial blood gases and S3-NIV scores between T0 and T1. Arterial blood gases were sampled from the radial artery in the morning, with patients breathing room air during spontaneous breathing.

**Figure 1:**
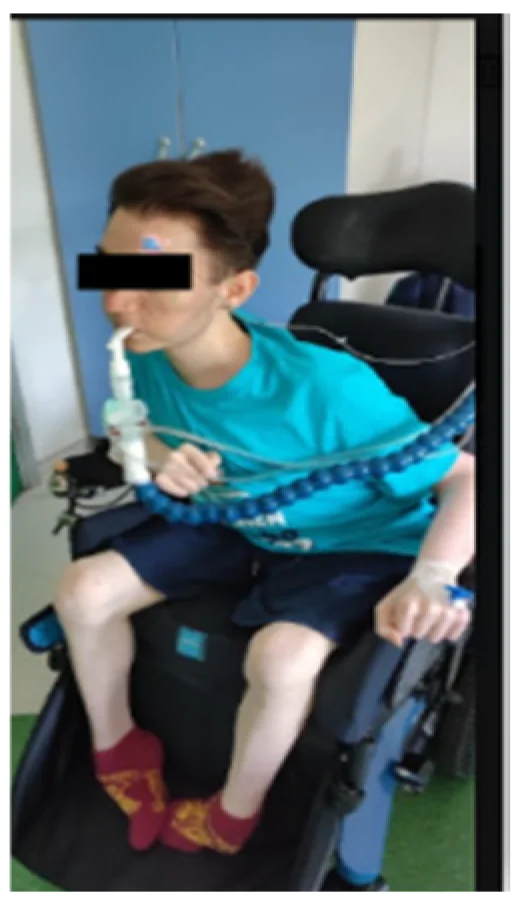
A patient with Duchenne muscular dystrophy using mouthpiece ventilation as non-invasive respiratory support.

All patients completed the S3-NIV questionnaire at the baseline assessment and at the follow-up visit (T1) 30 days later [22,23] (Figure 1).

### 2.5. Statistical Analysis

Given the pilot design, no formal sample size calculation was performed. Data are reported as individual values and mean ± standard deviation (SD). Changes between T0 and T1 are reported as mean paired differences with 95% confidence intervals (CI) and were tested with the exact Wilcoxon signed-rank test; with six patients, the smallest attainable two-sided *p* value is 0.031. Given the exploratory nature of the study, no correction for multiple comparisons was applied, and *p* values should be interpreted descriptively. Statistical analyses were performed using GraphPad Prism version 9.1.0 (GraphPad Software, San Diego, CA, USA).

## 3. Results

We performed a follow-up clinical assessment after 30 days to evaluate usage and efficacy. The six patients were aged 22–34 years (mean 26.8 ± 4.1 years) and had severe restrictive ventilatory impairment (FVC 0.67 ± 0.15 L) (Table 1). All patients tolerated MPV well. At T1, daytime ventilation was delivered by MPV instead of the nasal mask, nocturnal NIV was continued, and mean total daily ventilator use remained above 16 h. Blood gas parameters also improved, with a reduction in CO_2_ levels in all six patients, from 49.7 ± 7.1 to 43.5 ± 5.7 mmHg (mean paired difference −6.2 mmHg, 95% CI −8.2 to −4.1; *p* = 0.031), while PaO_2_ increased from 73.3 ± 6.0 to 81.3 ± 5.4 mmHg (+8.0 mmHg, 95% CI 4.8 to 11.2; *p* = 0.031); four of six patients were normocapnic at T1, whereas patients 1 and 2 remained hypercapnic (PaCO_2_ 49 and 52 mmHg), despite reductions of 6 and 9 mmHg, respectively. Patients reported improvement in pressure ulcers, where present, and a greater sense of psychological well-being because they could continue their activities of daily living. At baseline, S3-NIV questionnaire scores averaged 2.53 ± 0.69 points. After 30 days of combined MPV and nocturnal NIV use, a marked improvement was observed, with a mean score of 4.87 ± 0.91 points (mean paired difference +2.33, 95% CI 1.94 to 2.72; *p* = 0.031).

Table 1 shows the individual scores at baseline and T1.

## 4. Discussion

MPV is a valid option for patients who need respiratory support for many hours/day. Our patients were well adapted to nocturnal NIV. However, they reported disadvantages during the day due to significant interference with daily activities and skin lesions caused by prolonged use. The S3-NIV questionnaire is an easy and quick tool to evaluate the clinical assessment of outpatients with chronic respiratory failure initiated on long-term home NIV. Our patients reported improved comfort with respiratory support after 30 days of MPV.

Hypercapnia was not corrected in two patients (patients 1 and 2), although their PaCO_2_ decreased by 6 and 9 mmHg, respectively. These were the patients with the highest baseline PaCO_2_ (55 and 61 mmHg), and patient 2 also had the lowest FVC (0.44 L) and the longest daily ventilator use (>20 h/day), suggesting more advanced disease. Possible explanations include incomplete titration of ventilator settings within 30 days, the intermittent nature of MPV—disconnection periods have been associated with PaCO_2_ increases of more than 5 mmHg [24]—and the transient reduction in delivered tidal volume after each reconnection to the mouthpiece, which differs markedly among ventilators [25]. In such patients, ventilator settings require further optimization and close monitoring, and the efficacy of nocturnal ventilation should also be verified.

Other authors also reported that patients were satisfied with MPV and preferred the mouthpiece to the nasal mask; this may favour adherence to NIV [24]. They reported that disconnection time was the major limitation to NIV adherence. The authors reported that disconnection periods were associated with >5 mmHg increases in PaCO_2_ and >2% decreases in SpO_2_, but no clinical complications occurred before or after the monitoring period [24]. The most common type of asynchrony was ineffective effort, also suggesting a need for improved trigger sensitivity. The recently introduced MPV software, was provided by Lowenstein, ResMed and Philips. Each user was supplied with a specific ventilator model which allows insufflation to be triggered only by patient positioning and is an option for patients with severe muscle weakness [26]. Moreover, today, the software of many new ventilators is adding the mouthpiece mode [27].

Mouthpiece ventilation is used with single-limb, non-vented circuit ventilators in pressure-controlled or, more frequently, volume-controlled mode to allow air stacking. Volume-controlled ventilation is the mode usually chosen for MPV, as it allows breath stacking and guarantees the set volume despite changes in respiratory mechanics, e.g., in the presence of secretions, whereas pressure-targeted modes do not [25,26]. In our series, three patients were ventilated in a pressure-targeted mode for the reason given in the Methods; the sample does not allow a comparison between modes, and volume-controlled ventilation remains the preferred option. The patient can receive mouthpiece ventilation passively, using the ventilator’s set backup frequency, or actively trigger the breath, retaining part or all of the delivered volume. Either a simple single-tube circuit or a circuit with an exhalation valve can be used.

The valve is preferable for patients who cannot disconnect to exhale outside the circuit, because exhaling into a valveless circuit would cause rebreathing. Patients with the valve can stay connected for a long time in a row. The choice of circuit also determines the trigger strategy, which is particularly relevant in patients with DMD and weak inspiratory muscles. With a valveless circuit, dedicated MPV software can deliver the breath as soon as the lips seal the mouthpiece, through the interruption of a small bias flow (“kiss” trigger), without requiring the patient to generate a negative pressure or an inspiratory flow [25,26]; this minimizes ineffective efforts, the most frequent asynchrony during MPV [24], and makes a backup rate unnecessary, so that no breaths are delivered while the patient is disconnected and nuisance alarms are avoided [25,27]. With a valved circuit, this trigger is not available on some ventilators, and breaths are triggered by a conventional flow trigger, which requires an inspiratory effort; a backup rate then guarantees minimum ventilation in case of ineffective efforts, at the cost of timed breaths delivered into the room and possible alarms whenever the patient releases the mouthpiece, and of a patient–ventilator interaction closer to that of mask NIV [25,27]. In our series, the three patients with an open circuit used the dedicated MPV trigger without a backup rate, whereas the three patients with a valved circuit used a conventional flow trigger with a backup rate set slightly below their spontaneous breathing rate. The clinician should evaluate the patient’s ability to synchronize with the mouthpiece and to exhale outside the mouthpiece. Depending on neck mobility, the patient can keep the mouthpiece between their lips continuously or leave it for varying periods.

The patient can independently disconnect from the mouthpiece to speak, eat, cough or call a family member. MPV presents no risk of skin breakdown or conjunctivitis, does not cause claustrophobia, and has a lower risk of gastric distension. It is safer to permit glossopharyngeal breathing in the event of sudden ventilator failure or accidental disconnection from the ventilator [28]. Mouthpieces for daytime use may elicit salivation and, more rarely, vomiting [29], and long-term use can cause orthodontic deformities after 20 years of use. However, the same problem has been detected with traditional interfaces in pediatric patients. Nasal plugs or nose clips can prevent leakage through the nares in patients using lip-covering interfaces for mouthpiece NIV during sleep [26,27]. However, although rare, air may also be swallowed and cause gastric distension, and the same discomfort is reported with NIV. EPAP cannot be maintained for patients who use an open NIV system, and it is indeed rare that any of our patients require EPAP. When present, set apnea alarms at the highest threshold to avoid unnecessary activation and discomfort [24,26,27]. Because the patient repeatedly connects to and disconnects from the mouthpiece and leaks are unavoidable, MPV is technically demanding for home ventilators: bench studies have shown marked differences between devices, and between settings on the same device, in their ability to support MPV and to restore the delivered volume after reconnection [25,30]. Careful monitoring is always necessary during the adaptation phase because MPV requires true patient collaboration, and not all ventilators adapt quickly to the patient’s respiratory efforts [31,32].

However, due to its specific features and disadvantages (air leaks, etc.), MPV must be managed by expert hands and closely monitored. MPV use is likely limited to a few centres, given the time required to adapt patients to MPV and the need for close patient monitoring.

These findings are clinically relevant. Because survival in DMD now extends into the fourth decade [3,4,21], a growing number of adults require ventilatory support for more than 12 h/day, approximately 10% of the Italian DMD population [2]. In these patients, prolonged daytime mask ventilation frequently causes pressure ulcers, limits speech, eating and social participation, and may reduce adherence. MPV may allow continuous ventilatory support to be maintained without these drawbacks and may delay or avoid tracheostomy, as suggested by previous series [3,15,17]. The same approach may also benefit patients with other neuromuscular disorders requiring prolonged daytime ventilation, such as amyotrophic lateral sclerosis [32].

The main limitations of this study are the very small sample size and the short observation period. With only six patients, no conclusions on the efficacy of MPV can be drawn, and a 30-day follow-up allows only the assessment of feasibility and short-term tolerance; long-term adherence, disease progression, need for tracheostomy and survival could not be evaluated. Larger studies with longer follow-up are needed.

## 5. Conclusions

In this small series of patients with DMD on nocturnal NIV, daytime MPV was feasible, safe and well tolerated over 30 days and was associated with a reduction in PaCO_2_ and an improvement in S3-NIV scores, although hypercapnia persisted in two patients. Given the small sample and the short follow-up, these findings are preliminary; larger studies with longer follow-up are needed to confirm them and to define the role of MPV in the respiratory management of DMD.

## Figures and Tables

**Table 1:** Individual characteristics, arterial blood gases and S3-NIV scores at baseline (T0) and after 30 days of daytime MPV (T1).

	Age (Years)	FVC (L)	FEV1 (L)	PaO_2_ T0 (mmHg)	PaO_2_ T1 (mmHg)	PaCO_2_ T0 (mmHg)	PaCO_2_ T1 (mmHg)	pH T0	pH T1	Main Complaint with Daytime Mask NIV	Total NIV at T0 (h/Day)	S3-NIV T0	S3-NIV T1
Patient 1	26	0.66	0.60	69	77	55	49	7.35	7.43	Nasal pressure ulcer	>16	2.7	5.4
Patient 2	34	0.44	0.20	67	75	61	52	7.36	7.44	Nasal pressure ulcer	>20	1.8	4.1
Patient 3	22	0.78	0.69	71	78	44	39	7.41	7.46	Social interference	>16	1.8	3.6
Patient 4	28	0.56	0.44	72	84	49	41	7.47	7.42	Social interference	>16	3.4	6.1
Patient 5	27	0.75	0.72	78	88	47	42	7.45	7.44	Social interference	>16	2.3	4.8
Patient 6	24	0.82	0.78	83	86	42	38	7.38	7.40	Social interference	>16	3.2	5.2
Mean ± SD	26.8 ± 4.1	0.67 ± 0.15	0.57 ± 0.22	73.3 ± 6.0	81.3 ± 5.4	49.7 ± 7.1	43.5 ± 5.7	7.40 ± 0.05	7.43 ± 0.02	—	—	2.53 ± 0.69	4.87 ± 0.91

FVC: forced vital capacity; FEV1: forced expiratory volume in 1 s; PaO_2_ and PaCO_2_: arterial partial pressures of oxygen and carbon dioxide; NIV: non-invasive ventilation; Total NIV at T0: nocturnal NIV plus daytime nasal-mask ventilation; S3-NIV: score from 0 (greatest impact) to 10 (least impact); SD: sample standard deviation. Arterial blood gases were obtained under the conditions described in Section 2.4.

## Data Availability

The data presented in this study are available upon reasonable request from corresponding author.

## Abbreviations

The following abbreviations are used in this manuscript:

ABG	Arterial Blood Gas
CI	Confidence Interval
DMD	Duchenne Muscular Dystrophy
FEV1	Forced Expiratory Volume in 1 s
FVC	Forced Vital Capacity
MPV	Mouthpiece Ventilation
NIV	Non-Invasive Ventilation
PaCO2	Partial Pressure of Carbon Dioxide
PaO2	Partial Pressure of Oxygen
S3-NIV	Questionnaire on respiratory Symptoms, Sleep and Side effects of NIV
SD	Standard Deviation
VC	Vital Capacity
